# Anti-kindling effect of *Ginkgo biloba* leaf extract and L-carnitine in the pentylenetetrazol model of epilepsy

**DOI:** 10.1007/s11356-022-19251-6

**Published:** 2022-02-22

**Authors:** Amina E. Essawy, Soad Ahmed El-Sayed, Ehab Tousson, Horeya S. Abd El-gawad, Reem Hasaballah Alhasani, Heba-Tallah Abd Elrahim Abd Elkader

**Affiliations:** 1 Zoology Department, Faculty of Science, Alexandria University, Alexandria, Egypt; 2 Zoology Department, Faculty of Science, Tanta University, Tanta, Egypt; 3 Department of Biology, Faculty of Applied Science, Umm Al-Qura University, Makkah, Saudi Arabia; 4 Zoology, Biological and Geological Sciences Department, Faculty of Education, Alexandria University, Alexandria, Egypt

**Keywords:** Epilepsy, Pentylenetetrazol, *Ginkgo biloba*, L-carnitine, Oxidative stress, Monoamine neurotransmitters, EZR-ir

## Abstract

Epilepsy is one of the most common serious brain disorders, affecting about 1% of the population all over the world. *Ginkgo biloba* extract (GbE) and L-carnitine (LC) reportedly possess the antioxidative activity and neuroprotective potential. In this report, we investigated the possible protective and therapeutic effects of GbE and LC against pentylenetetrazol (PTZ)-induced epileptic seizures in rat hippocampus and hypothalamus. Adult male albino rats were equally divided into eight groups: control, GbE (100 mg/kg), LC (300 mg/kg), PTZ (40 mg/kg), protective groups (GbE + PTZ and LC + PTZ), and therapeutic groups (PTZ + GbE and PTZ + LC). The oxidative stress, antioxidant, and neurochemical parameters, viz., malondialdehyde (MDA), nitric oxide (NO), reduced glutathione (GSH), superoxide dismutase (SOD), catalase (CAT), glutathione peroxidase (GPx), acetylcholine esterase (AchE), dopamine (DA), norepinephrine (NE), and serotonin (5-HT), in the hippocampal and hypothalamic regions have been evaluated. PTZ injection leads to an increase in the seizure score, the levels of MDA and NO, and to a decrease in the activity of GSH, SOD, CAT, and GPx. Besides, monoamine neurotransmitters, DA, NE, and 5-HT, were depleted in PTZ-kindled rats. Furthermore, PTZ administration caused a significant elevation in the activity of AchE. Hippocampal and hypothalamic sections from PTZ-treated animals were characterized by severe histopathological alterations and, intensely, increased the ezrin immunolabeled astrocytes. Pre- and post-treatment of PTZ rats with GbE and LC suppressed the kindling acquisition process and remarkably alleviated all the aforementioned PTZ-induced effects. GbE and LC have potent protective and therapeutic effects against PTZ-induced kindling seizures via the amelioration of oxidative/antioxidative imbalance, neuromodulatory, and antiepileptic actions.

## Introduction

Epilepsy is a common neurological disease that influences approximately 1% of humans worldwide with a much higher incidence in developing countries (Mohamed and Eltony 2020). Clinically, it is a dynamic and chronic disorder of abnormal neuronal electrical activity, characterized by recurrent unprovoked seizures through an imbalance between excitability and inhibition in the brain which may either affect specific brain systems or originate in a restricted area and spread to involve multiple cortical and subcortical circuits (Akyuz et al. 2020; Wu et al. 2020). The mechanism of epileptogenesis is still unclear; however, the pathophysiology of epilepsy might include oxidative stress, neuroinflammation, and neurotransmitter system dysfunction (Wu et al. 2020). Individuals with epilepsy are characterized by the progressive deterioration of neurological functions, psychosocial disorders, and systemic diseases (Zhu et al. 2020; Badawi et al. 2021).

Although many antiepileptic drugs have been developed, most of them have no effect on the cognitive impairment of refractory epilepsy. Moreover, these drugs also cause multiple side effects, including depressive mood, psychosis, increased irritability, and aggressive behavior (Hixson 2014; Wang et al. 2021). Therefore, there is urgent research to develop safer and effective antiepileptic drugs, especially for refractory epilepsy (Wang et al. 2021). Recently, many herbal products have been illustrated to have anticonvulsant activity. Among these herbal products are *Ginkgo biloba* extract (GbE) and L-carnitine (Salama et al. 2015; El-sendiony et al. 2020).

*Ginkgo biloba*, a living fossil, belongs to the Ginkgoaceae family and has been used as an herbal remedy for many centuries in China (Chen et al. 2020). GbE, a traditional Chinese herbal medicine, has a long history of medical use for treating cerebral dysfunction, brain aging, and neurodegenerative dementia (Abdou et al. 2016). GbE has a wide neuroprotective effect which is determined by the following active constituents: flavonol glycosides (quercetin, kaempferol, and isorhamnetin) and terpene trilactones (ginkgolides and bilobalide), proanthocyanidins, alkylphenols, biflavones, simple phenolic acids, 6-hydroxykynurenic acid, 4-O-methylpyridoxine, and polyprenols (Chen et al. 2020; Mohammed et al. 2020). Numerous studies have verified that GbE has pharmacological modes of action including antioxidant and antiapoptotic effects (Shahidi et al. 2021).

L-Carnitine (LC), a trimethylamine-β-hydroxybutyrate, is produced naturally in the body from the amino acids’ methionine and lysine (Ali 2020; Sallam et al. 2021). LC is an essential mitochondrial respiratory cofactor needed for the transport of fatty acids into the mitochondrial matrix during the generation of metabolic energy (Aboubakr et al. 2020). Salimi et al. and Elkomy et al. (2020) mentioned that LC plays a crucial role in oxidative/antioxidative balance and has an antiperoxidative action on several animal tissues**.**

Pentylenetetrazol (PTZ), a γ-aminobutyric acid (GABA)ergic receptor antagonist, has a stimulant epileptogenic property and is commonly used as a convulsing drug in experimental studies. A tonic–clonic seizure was developed by a single or repeated injection of PTZ which causes cognitive deficit and a gradual decrease in short-term memory function (Mohamed and Eltony 2020).

The current study aimed to investigate the protective and therapeutic potentials of GbE and LC in the PTZ rat model of epileptic seizures.

## Materials and methods

### Chemicals

Pentylenetetrazol (PTZ) of the highest purity was obtained from Sigma-Aldrich Corporation (Sigma-Aldrich, St. Louis, USA). Standardized leaf extract of *Ginkgo biloba* (EGb 761) (glycosides, 24%, and terpene lactones, 6%) and L-carnitine (LC) was purchased from Arab Company for Pharmaceuticals and Medical Plants (Egypt). All other reagents and kits used in this study were purchased from the Biodiagnostic Company (Egypt). PTZ, EGb 761, and LC were dissolved in 0.9% physiological saline at use.

### Animals

Male *Wistar* albino rats (180–200 g) were purchased from Medical Research Institute, Alexandria University, Egypt. They were housed in cages under the regulated conditioning room at 25 ± 1 °C, 50 ± 5% relative humidity with 12-h light/dark cycles and free access to basal diet and water. They were acclimatized for 1 week before the experiment with monitoring of the body weight and general conditions. The animal handling and care methods were strictly approved by the NIH principles and the guidelines of the Institutional Animal Care and Use Committee (IACUC) (protocol approval code: AU 04,210,224,303), Alexandria University, Egypt.

### Pentylenetetrazol-kindled rat model and scoring of epileptic seizures

For induction of epileptic seizures, we used the method previously described by Hansen et al. (2016). For kindling a dose of 40 mg/kg/b.w, PTZ was injected intraperitoneal (i.p.) once every 48 h for 17 days until the animal exhibited full motor seizures. After each PTZ injection, each rat was observed for 30 min for latency to an epileptic fit, duration of seizure, and seizure stage according to a modified Racine scale (Giardina and Gasior 2009) as follows: stage 0, no response; stage 1, ear and facial twitching; stage 2, convulsive waves axially through the body; stage 3, myoclonic body jerks; stage 4, turn over into side position, clonic–tonic seizures; and stage 5, turn over into back position, generalized tonic–clonic seizures, and/or mortality. Complete kindling was defined as exhibiting stage 4 or 5 of seizure score on 3 consecutive trials.

### Experimental protocol

A total of eighty rats were assigned at random into 8 groups (10 rats/ group) and treated every 48 h as follows: control group received only the vehicle (saline solution); GbE group was given orally 100 mg/kg EGb (Rodriguez de Turco et al.^1993^); LC group received oral gavage treatment 300 mg/kg LC (Tousson et al. 2014); PTZ group was given intraperitoneally 40 mg/kg (Waggas and Al-Hasani 2010); GbE + PTZ protective group was given 100 mg/kg EGb for 17 days, followed by 40 mg/kg PTZ for other 17 days; LC + PTZ protective group was given 300 mg/kg LC for 17 days, followed by 40 mg/kg PTZ for other 17 days; PTZ + GbE therapeutic group was given 40 mg/kg PTZ for 17 days, followed by 100 mg/kg EGb for other 17 days; and PTZ + LC therapeutic group was given 40 mg/kg PTZ for 17 days, followed by 300 mg/kg LC for other 17 days.

After the 5-week experimentation period, the rats were euthanized with sodium thiopental (1%, 30 mg/kg intravenously during 30 s) and decapitated by cervical dislocation. The brain of 6 rats from each group was rapidly removed, washed with cold 0.9% NaCl, and dissected to isolate the hippocampus and hypothalamus regions for the assessment of biochemical parameters. The tissue of the two brain regions was homogenized (10% w/v) in an ice-cold buffer containing 1.15% KCl and 0.01 M sodium phosphate buffer (pH 7.4) per gram tissue. Moreover, the hippocampus and hypothalamus of 4 rats from each group were dissected out, fixed in 4% paraformaldehyde, and used for histological and immunohistochemical investigations.

### Assay of oxidative stress-related markers

The lipid peroxidation end product (malondialdehyde, MDA) was measured by the thiobarbituric acid method (Ohkawa et al. 1979). The nitric oxide (NO) level in brain supernatant was determined according to Koracevic et al. (2001) protocol. The level of reduced glutathione (GSH) was assayed as described by Jollow et al. (1974). The enzymatic activities of the antioxidants SOD, GPx, and CAT were calculated through the previously procedures reported by Yousef et al. (2020).

### Assessment of neurochemical biomarkers

**Fig. 1:**
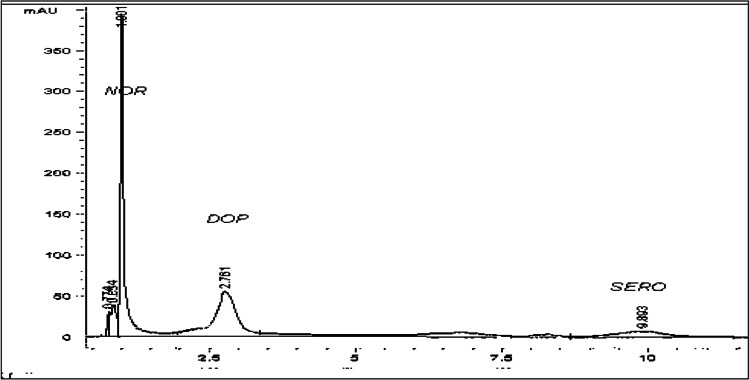
The chromatogram of the monoamine’s standard separated by HPLC

The method described by Ellman et al. (1961) was applied to assay the activity of acetylcholine esterase (AchE). Enzyme activity was determined using a molar extinction coefficient of 412 nm. The dopamine (DA), serotonin (5-HT), and norepinephrine (NE) levels in the hippocampus and hypothalamus tissue were assayed using HPLC as previously described by Hegazi and Hasanein (2010). Brain tissues were weighed and homogenized in 10% w/v of HPLC high-grade methanol. The brain homogenates were centrifuged at 3000 rpm for 10 min and rapidly extracted from the lipids and trace elements using solid-phase CHROMABOND extraction column (cat. No. 730031). The sample was then injected directly to the AQUA column (150 mm × 4.6 mm, 5 µ, and C18) under the following conditions: mobile phase 97/3 20 mM potassium phosphate, pH = 3, flow rate 1.5 ml/min, and UV 270 nm. Based on the chromatogram peak areas (Fig. 1), the levels of monoamines were calculated using the calibration curve of the peak area versus concentration by Eurochrom HPLC software, version 1.6, and expressed as µg/g brain tissue.

### Histopathological investigation

Paraformaldehyde-fixed hippocampus and hypothalamus were hydrated within increasing concentrations of ethanol and then cleared in xylene and embedded in paraffin wax according to standard procedures. Paraffin Sects. (5 μm thick) were mounted and stained with hematoxylin and eosin for studying the histopathological changes according to Suvarna et al. (2018). The severity of structural changes was estimated quantitatively using ImageJ analyzer (version 5) in 5 visual fields in 3 sections of 4 animals in each group according to Sakr et al. (2011).

### Immunohistochemical detection of ezrin

Paraffin sections of the hippocampus and hypothalamus from different groups were used for ezrin immunohistochemical staining. The expression of ezrin proteins was detected using avidin–biotin complex (ABC) method (Ramprasad et al. 1996). Deparaffinized and rehydrated sections were rinsed in distilled water for 5 min, washed in PBST for 10 min, and incubated with 10% normal goat serum for 15 min to reduce non-specific background staining. Then, the sections were incubated with anti-rabbit ezrin (Dako, 1:100) for 1–2 h at room temperature. The sections after 5 baths in PBST were incubated with biotinylated goat anti-rabbit immunoglobulin (Nichirei, Tokyo, Japan) for 1 h at ambient temperature. The sections were incubated with 3–3’-diaminobenzidine (DAB, Wako pure chemical industries, Ltd) for 7–9 min in dark at room temperature. Negative control sections were done by substituting ezrin primary antibodies by normal serum of rabbit. The prepared sections were examined by means of a research microscope and analyzed for the positive ezrin-immunostaining using the ImageJ analyzer (version 5.1).

### Statistical analysis

Statistical analysis was analyzed using SPSS, version 22, and all data were expressed as mean ± SE. Statistical significance differences in data were determined using one-way ANOVA followed by post hoc least significant difference (LSD) for comparison of each two means. A *p* value of less than 0.05 was regarded as a significant difference.

#### Results

### Kindling score

**Fig. 2:**
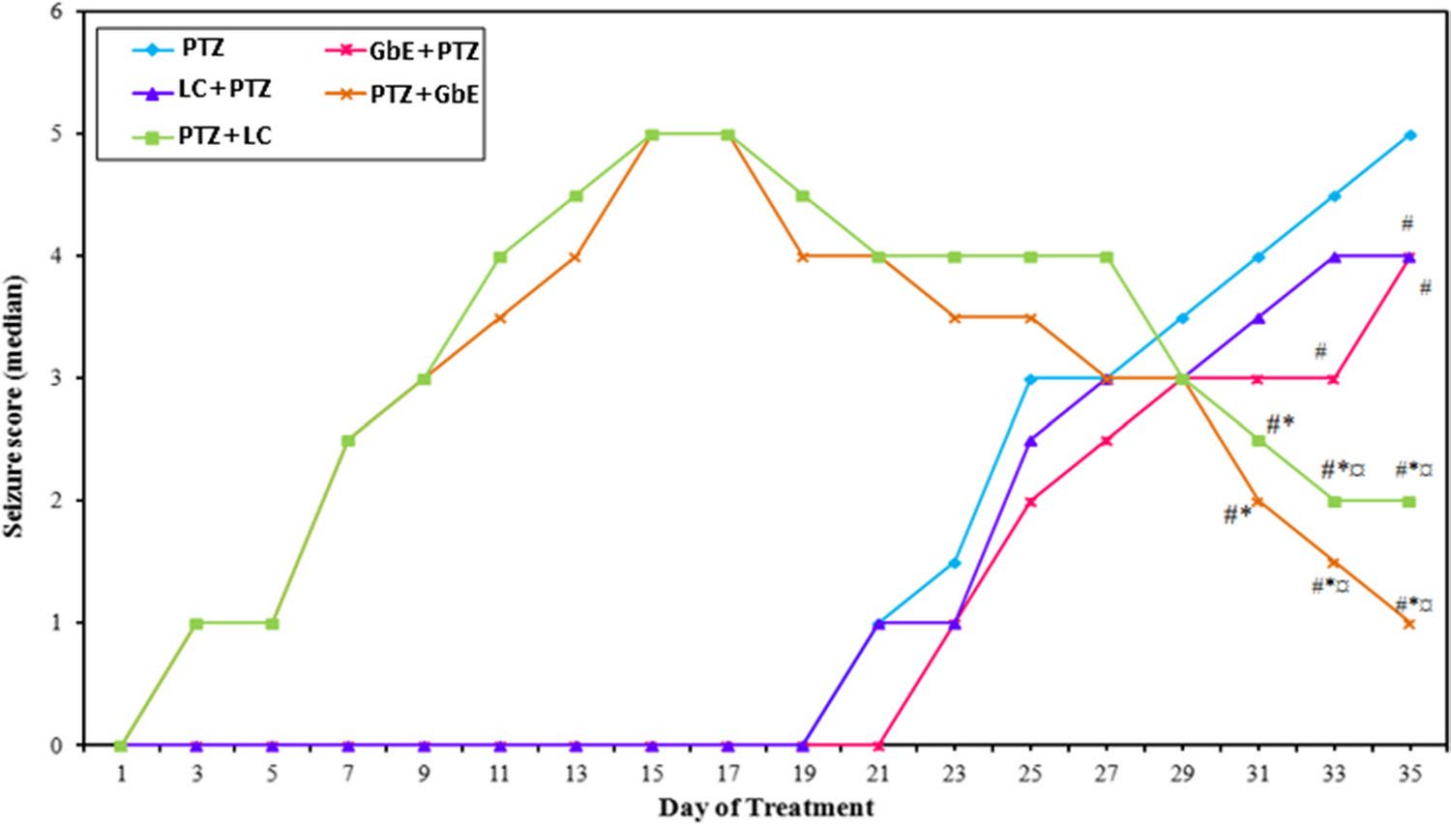
Effects of *Ginkgo biloba* leaf extract (GbE) and L-carnitine (LC) pre- and post-treatment on the development of pentylenetetrazol (PTZ)-kindled seizures. Data are expressed as median seizure score. ^**#**^ Significant with PTZ,^*^ significant with GbE + PTZ, and ^**¤**^ significant with LC + PTZ at *p* ≤ 0.05

Repeated administration of a sub-convulsant dose of PTZ resulted in increasing convulsive activity that include hyperactivity, twitching, and hyperextension of the limbs and further progressed to a generalized tonic–clonic seizure score of 5. On the other hand, treatment with GbE or LC significantly attenuated the severity of kindling as evidenced by a reduction in kindling score as compared with PTZ-treated animals (Fig. 2). Administration of GbE before PTZ injection significantly decreased (*p* ≤ 0.05) the seizure score on days 33 and 35. However, administration of LC before PTZ injection significantly decreased the seizure score on day 35 only. Rats treated with GbE or LC after PTZ injection showed decreases (*p* ≤ 0.05) in median seizure scores on days 31, 33, and 35 compared to the PTZ-kindled group.

### Effect of GbE and LC on oxidative stress and antioxidant markers in the hippocampus and hypothalamus of PTZ-treated rats

**Fig. 3:**
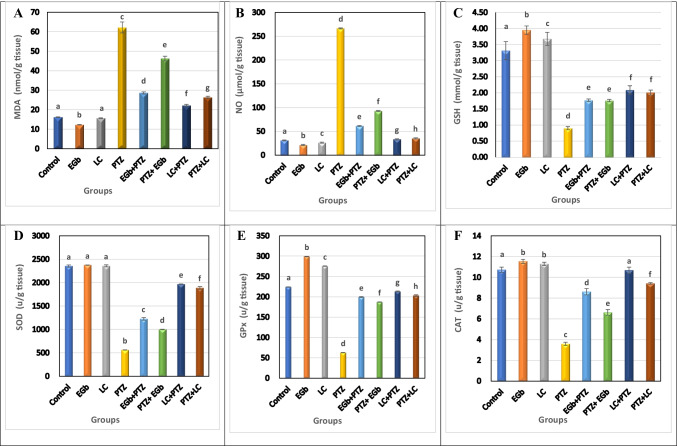
Effect of *Ginkgo biloba* leaf extract (GbE) and L-carnitine (LC) on the level of **A** malondialdehyde (MDA), **B** nitric oxide (NO), **C** reduced glutathione (GSH), **D** superoxide dismutase (SOD), **E** glutathione peroxidase (GP_X_), and **F** catalase (CAT) in the hippocampus of rats treated with pentylenetetrazol (PTZ). All the data were analyzed using one-way ANOVA followed by LSD post hoc test. Values are expressed as mean ± SE; *n* = 6 rats for each group. Different superscripts on the columns are significantly different at *p* ≤ 0.05

**Fig. 4:**
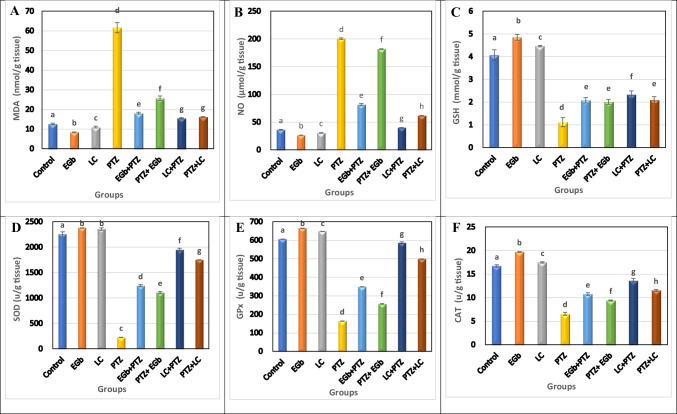
Effect of *Ginkgo biloba* leaf extract (GbE) and L-carnitine (LC) on the level of **A** malondialdehyde (MDA), **B** nitric oxide (NO), **C** reduced glutathione (GSH), **D** superoxide dismutase (SOD), **E** glutathione peroxidase (GP_X_), and **F** catalase (CAT) in the hypothalamus of rats treated with pentylenetetrazol (PTZ). All the data were analyzed using one-way ANOVA followed by LSD post hoc test. Values are expressed as mean ± SE; *n* = 6 rats for each group. Different superscripts on the columns are significantly different at *p* ≤ 0.05

Figures 3 and 4 show that treatment with PTZ resulted in a significant (*p* ≤ 0.05) increase in the level of oxidative stress markers, MDA and NO, and a significant decrease in the activity of GSH and the enzymatic antioxidants (SOD, GPx, CAT) in the hippocampus and hypothalamus of animals treated with PTZ compared to controls.

The level of MDA and NO was found to be significantly inhibited in both protective (GbE + PTZ and LC + PTZ) and therapeutic (PTZ + GbE and PTZ + LC) groups compared to PTZ-treated animals. However, the reduction of MDA and NO levels was more pronounced in therapeutic groups. Also, supplementation with GbE or LC (before or after PTZ administration) significantly improved the measured antioxidant markers. The elevation of the antioxidant’s activity was more pronounced in therapeutic treatment than in the protective one. Moreover, GbE administration significantly enhanced the antioxidants activity more than LC administration.

### Effect of GbE or LC on the level of DA, NE, 5-TH, and AchE in the hippocampus and hypothalamus of PTZ-treated rats

**Fig. 5:**
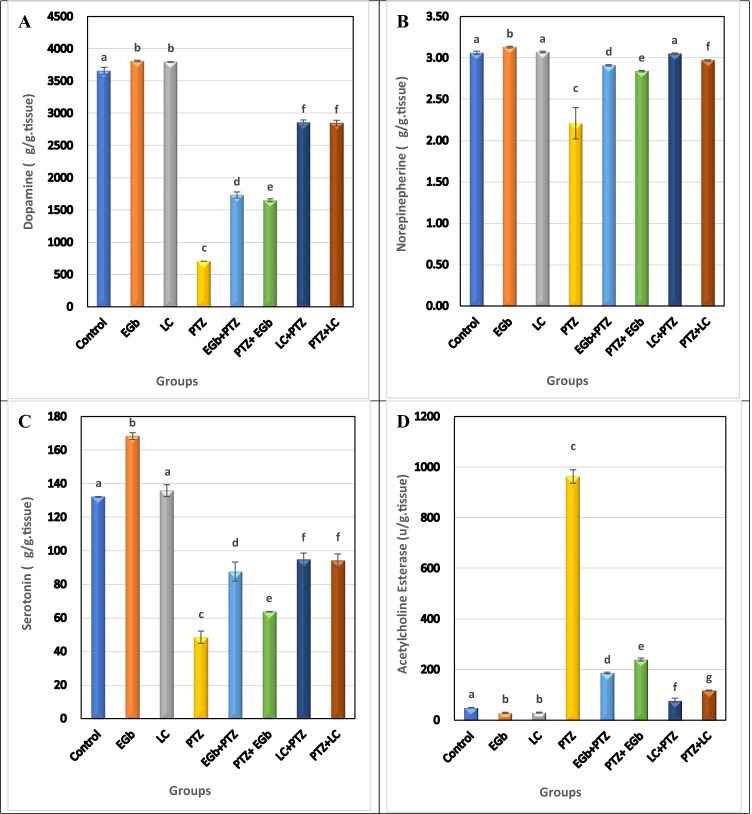
Effect of *Ginkgo biloba* leaf extract (GbE) and L-carnitine (LC) on the level of **A** dopamine (DA), **B** norepinephrine (NE), **C** serotonin (5-TH), and **D** acetylcholine esterase (AchE) in the hippocampus of rats treated with pentylenetetrazol (PTZ). All the data were analyzed using one-way ANOVA followed by LSD post hoc test. Values are expressed as mean ± SE; *n* = 6 rats for each group. Different superscripts on the columns are significantly different at *p* ≤ 0.05

**Fig. 6:**
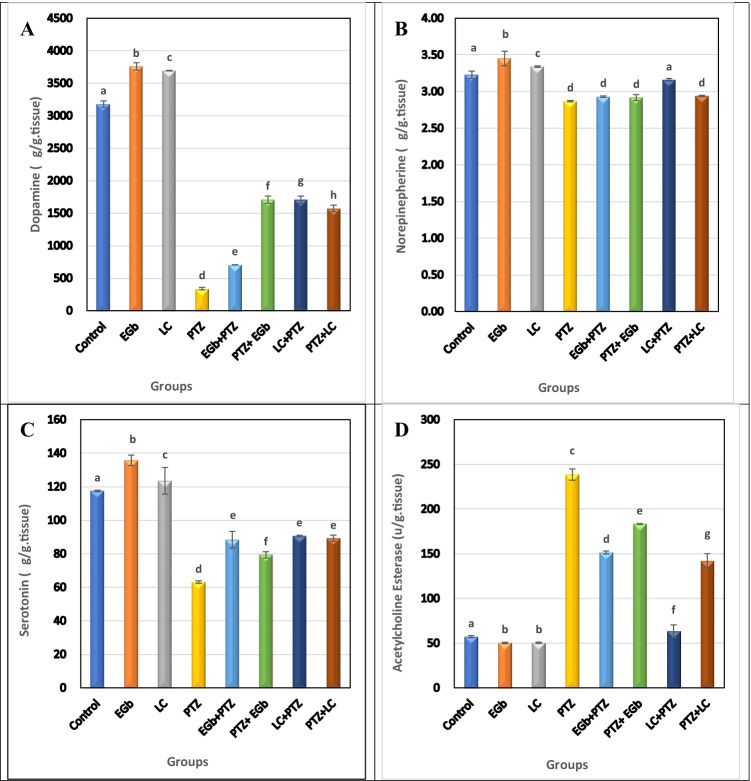
Effects of *Ginkgo biloba* leaf extract (GbE) and L-carnitine (LC) on the level of **A** dopamine (DA), **B** norepinephrine (NE), **C** serotonin (5-TH), and **D** acetylcholine esterase (AchE) in the hypothalamus of rats treated with pentylenetetrazol (PTZ). All the data were analyzed using one-way ANOVA followed by LSD post hoc test. Values are expressed as mean ± SE; *n* = 6 rats for each group. Different superscripts on the columns are significantly different at *p* ≤ 0.05

The present results showed that PTZ significantly (*p* ≤ 0.05) decreased the level of DA, NE, and 5-HT and increased the activity of AchE in the hippocampus and hypothalamus compared to control groups (Figs. 5 and 6).

On the other hand, protective and therapeutic groups treated with GbE or LC showed a significant increase in DA, NE, and 5-HT levels and a significant decrease in AchE activity versus PTZ-treated animals in both the tested brain regions. However, a non-significant elevation in NE level was recorded in the hypothalamus of protective groups. The improvement in the level of the tested neurotransmitters and AchE was more pronounced in the therapeutic groups than in the protective ones.

### Histopathological observations

**Fig. 7:**
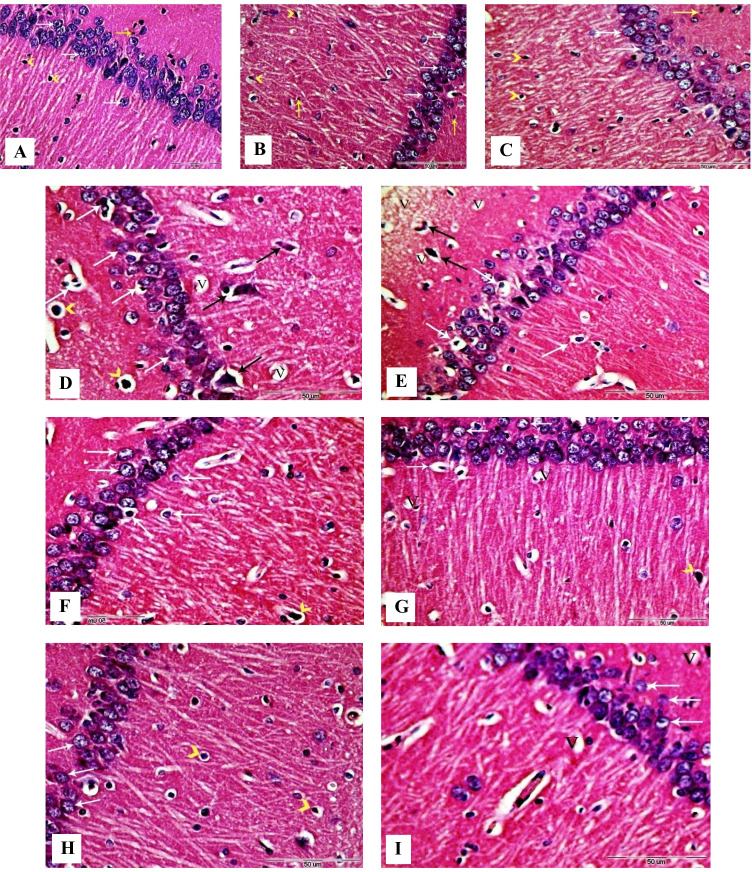
Photomicrographs of coronal sections in the hippocampus of male rats. **A** Section from control rat, showing normal structure of the hippocampus with pyramidal cells (white arrows), astrocytes (arrowheads), and microglial cells (yellow arrow). **B**, **C** Sections from GbE and LC-treated rats respectively, showing normal structure of the hippocampus with normal histology of pyramidal cells (white arrows), astrocytes (black arrows), and microglial cells (yellow arrows). **D**, **E** Sections from PTZ-treated rats, showing large number of degenerated neurons with pyknotic and shrunken nuclei (white arrows), numerous hyper-chromatic astrocytes with vacuolated cytoplasm (black arrows), and hyper-chromatic microglial cells (arrowheads). **F** Section from GbE + PTZ-treated rat, showing some neurons with pyknotic and (white arrows) and astrocytes with vacuolated cytoplasm (arrowhead). **G** Section from LC + PTZ-treated rat, showing few neurons with chromatolysis or shrunken nuclei (white arrows) and some hyper-chromatic astrocytes (arrowhead). **H** Section from PTZ + GbE-treated rat, showing pyramidal neurons with normal appearance (white arrows) and few hyper-chromatic astrocytes with vacuolated cytoplasm(arrowheads). **I** Section from PTZ + LC-treated rat, showing a few numbers of neurons with mild diffuse vacuolar degeneration (white arrows). V, vacuoles (H&E, X400)

Quantitative analysis of histopathological structural changes of the hippocampus and hypothalamus was recorded in Table 1. Coronal sections in the hippocampus of control, GbE, and LC groups (Fig. 7A, B, C) showed the presence of normal mature pyramidal neurons with rounded vesicular nuclei and prominent distinct centric nucleoli. Also, numerous astrocytes, microglia, and organized neuropil were observed with normal structure.

**Table 1:** Quantitative assessment of hippocampus and hypothalamus structural changes in rats after 5 weeks of treatment

	**Control**	**GbE**	**LC**	**PTZ**	**GbE + PTZ**	**PTZ + GbE**	**LC + PTZ**	**PTZ + LC**
**Hippocampus** Normal cells Vacuolation Astrocytes + microglia	13.20 ^c^ ± 0.51 1.70 ^a^ ± 0.12 3.60 ^bc^ ± 0.29	7.60^a^ ± 0.43 2.60 ^b^ ± 0.19 2.60 ^a^ ± 0.19	10 ^b^ ± 0.22 2.60 ^b^ ± 0.19 3.20 ^ab^ ± 0.25	8.60 ^a^ ± 0.43 15.60 ^f^ ± 0.19 7.20 ^e^ ± 0.34	7.60 ^a^ ± 0.43 4.40 ^d^ ± 0.19 4.40 ^d^ ± 0.19	13.60 ^a^ ± 0.43 5 ^e^ ± 0.27 4 ^bcd^ ± 0.16	8 ^c^ ± 0.27 5.20 ^e^ ± 0.12 3.80 cd ± 0.25	10 ^b^ ± 0.22 3.80 ^c^ ± 0.25 3.80 ^bcd^ ± 0.25
**Hypothalamus** Normal cells Vacuolation Astrocytes + microglia	23.20 ^f^ ± 0.25 1 ^a^ ± 0.00 4.20 ^c^ ± 0.12	15.40 ^c^ ± 0.19 1.60 ^b^ ± 0.10 2 ^a^ ± 0.16	13.60 ^b^ ± 0.43 1.70 ^b^ ± 0.12 3.20 ^b^ ± 0.25	8.80 ^a^ ± 0.25 8.40 ^e^ ± 0.19 6.20 ^e^ ± 0.25	18.20 ^d^ ± 0.25 4.40 ^c^ ± 0.19 5.60 ^de^ ± 0.19	14.40 g ± 0.40 5.80 ^c^ ± 0.12 5 ^e^ ± 0.27	26 ^c^ ± 0.35 2.40 ^d^ ± 0.19 5.80 ^d^ ± 0.12	20.40 ^e^ ± 0.19 1.30 ^ab^ ± 0.12 3.80 ^bc^ ± 0.25

Data were analyzed using one-way ANOVA followed by LSD post hoc test. Values are expressed as mean ± SE; *n* = 4 rats for each group. Different superscripts on the columns are significantly different at *p* ≤ 0.05

Brain coronal sections from PTZ-treated rats (Fig. 7D and E) revealed a loosely packed pyramidal cell layer and numerous histopathological changes including neuronal atrophy, degenerated pyramidal cells, and vacuolated neurocytes. Nuclei of the cells were shrunken, pyknotic, and hyper-chromatic. Also, many degenerated apoptotic astrocytes and microglia were seen with pyknotic nuclei.

The hippocampus coronal sections from protective groups treated with GbE or LC (Fig. 7F and G, respectively) revealed minor histopathological changes represented by diffuse vacuolation, some damaged neurons, and a few apoptotic astrocytes. In therapeutic groups treated with GbE or LC (Fig. 7H and I, respectively), an improvement in the neuronal structure with minimal distortion of the pyramidal cells and few hyper-chromatic astrocytes was observed.

**Fig. 8:**
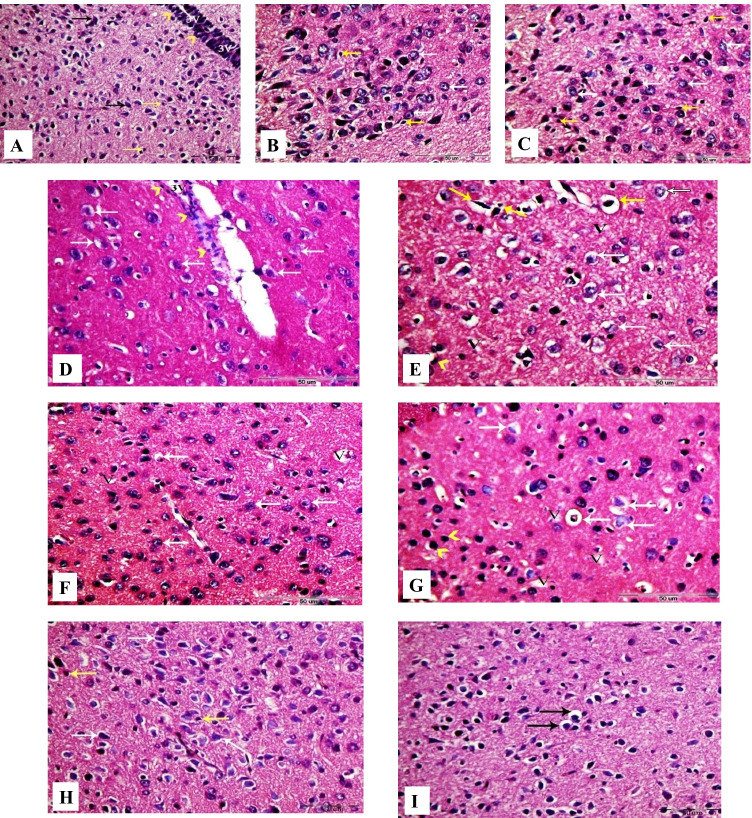
Photomicrographs of coronal sections in the hypothalamus of male rats. **A** Section from control rat, showing normal structure of the hypothalamus neurons (black arrows) and astrocytes (yellow arrows). Arrowheads indicate ependymal cells of the third ventricle (3 V). **B**, **C** Sections from GbE and LC-treated rats, respectively, showing normal hypothalamus neurons (white arrows) and astrocytes (yellow arrows). **D**, **E** Sections from PTZ-treated rats, showing large number of degenerated neurons (white arrows) and diffuse vacuolar degeneration (V). Arrowheads point at scattered ependymal cells of the third ventricle (3 V) and hyper-chromatic astrocytes with vacuolated cytoplasm (yellow arrows). **F** Section from GbE + PTZ-treated rat, showing mild diffuse vacuolar degeneration (V) and mild number of degenerated neurons (white arrows). **G** Section from LC + PTZ-treated rat, showing few neurons with diffuse vacuolar degeneration (V), some degenerated neurons (white arrows), and some hyper-chromatic microglial cells (arrowheads). **H** Section from PTZ + GbE-treated rat, showing more or less normal neurons (white arrows) and normal astrocytes (yellow arrows). **I** Section from PTZ + LC-treated rat, showing a few numbers of neurons with shrunken nuclei and perinuclear vacuolization (black arrows) (H&E, X400)

Rat hypothalamic sections from control, GbE, and LC groups showed normal histological structures of the neurons and nerve fibers (Fig. 8A, B, and C).

However, sections from PTZ-treated rats (Fig. 8D and E) showed diffuse vacuolar degeneration and a large number of degenerating shrunken neurons. In addition, mild spongy changes in hypothalamic nuclei were noticed, and the ependymal cell layer of the third ventricle is loosely packed and appeared scattered.

Hypothalamus coronal sections from GbE + PTZ and LC + PTZ-treated groups (Fig. 8F and G, respectively) revealed mild tissue injury with few vacuolar degenerations. Furthermore, hypothalamus coronal sections from PTZ + GbE group (Fig. 8H) showed better-organized structures with well-established neurons which resembled that of the control groups. On the other hand, PTZ + LC group (Fig. 8I) revealed a few numbers of damaged neurons with mild vacuolar degeneration.

### Immunohistochemical observation

**Fig. 9:**
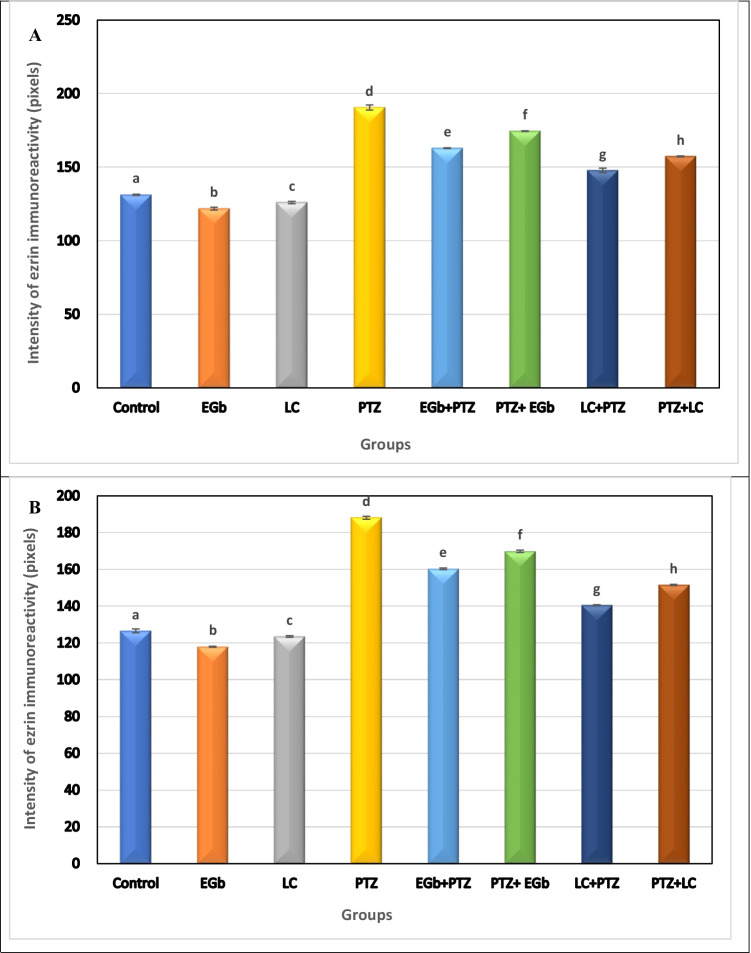
Effects of *Ginkgo biloba* leaf extract (GbE) and L-carnitine (LC) on the expression of ezrin immunoreactivity in the hippocampus (**A**) and hypothalamus (**B**) of rats treated with pentylenetetrazol (PTZ). All the data were analyzed using one-way ANOVA followed by LSD post Hoc test. Values are expressed as mean ± SE; *n* = 6 rats for each group. Different superscripts on the columns are significantly different at *p* ≤ 0.05

To demonstrate the involvement of astroglial activation in PTZ-induced epileptic seizures and the effect of GbE and LC on the activation of these cells, the intensity of ezrin immunoreactivity (EZR-ir) was shown in the hippocampus and hypothalamus of the different groups (Fig. 9A and B, respectively). In both regions, EZR-ir expression was significantly elevated in PTZ-treated rats as compared with all other groups suggesting the activation of astroglial cells. Furthermore, the intensity of EZR-ir immunostaining in both tested brain regions showed a marked decrement in rats treated with GbE compared with rats treated with LC. Also, the intensity of EZR-ir in astroglia was significantly decreased in rats treated with PTZ followed by GbE or LC (therapeutic groups) compared with rats treated with GbE or LC followed by PTZ (protective groups).

## Discussion

Epilepsy is a complex prevalent neurobehavioral disorder affecting 50 million of the world’s total population. In the present study, sequential injections of sub-convulsive dose of PTZ induced successively evoked chemical kindling in rats as evident from the seizure score. This finding agrees with previous studies carried out by Hamdy et al. (2020) and Badawi et al. (2021) who illustrated that the long-lasting seizure-evoked neuronal hyperactivity could result in an irreversible deleterious neurological change that finally ends by neuronal degeneration. The recurrence of seizures is associated with decreased numbers of GABA_A_ receptor chloride channels, which attenuates GABA-dependent inhibition and/or activation of N-methyl-D-aspartate (NMDA) receptors (Ali et al. 2004; Ceyhan et al. 2005) and amplification in glutamate release (Rodrigues et al. 2012).

However, treatment with GbE and LC significantly attenuated the elevated signs of seizure severity score in kindled animals, which indicates their protective effect against PTZ-induced seizures. GbE and LC exhibit numerous pharmacological effects such as antioxidative, anti-inflammatory, and antiapoptotic properties that are associated with their neuroprotection (Hamza et al. 2020; Shahidi et al. 2021). This finding is in line with Ahmed and Mahmoud (2012) who found that GbE and LC inhibit seizures and increase the anticonvulsant activity induced by PTZ.

Oxidative stress and neuroinflammation co-exist in the brains of patients with different types of epilepsy. NF-κB was found to be a factor switching an inflammatory state after oxidative stress (Borowicz-Reutt and Czuczwar 2020). High level of oxidative stress causes increased IL-1β and toll-like receptor 4 (TLR4) expression which reinforces NF-κB signaling further. In this case, a regulatory protein SOCS-1 cannot balance, creating a vicious circle of a self-sustaining inflammatory signal. Oxidative stress could accelerate the development of epilepsy and exhibit reciprocal causation in epileptogenesis. In the current study, a marked elevation in MDA levels was recorded in the hippocampus and hypothalamus in PTZ-kindled animals. This result is in accordance with the previous studies that showed lipid peroxidation increment in the brain during epileptic seizures (Alachkar et al. 2020; Hamdy et al. 2020).

NO is a molecule that is associated with the regulation of neuronal stimulation ability and epileptic activity. Consistent with a previous report (Yuan et al. 2020), our results indicated a significant elevation in the brain NO in PTZ-administered animals. NO exerts an epileptogenic effect by increasing glutamate activity through enhanced cyclic guanosine monophosphate (cGMP) levels (Kurt et al. 2016).

The antioxidant system including GSH, SOD, GPx, and CAT play a vital role as a free radical scavenger to protect cells against oxidative stress (Aboubakr et al. 2021). The current results showed that PTZ potentiated a state of oxidative/antioxidative imbalance in the hippocampal and hypothalamic tissues as represented by the decreased levels of GSH and reduced the enzymatic activities of SOD, GPx, and CAT. These results are in parallel to what has been previously shown (Alachkar et al. 2020; Yuan et al. 2020). Depletion of GSH content and antioxidant enzyme activity in brain tissue is associated with epileptic seizures in human and animal models due to the excessive generation of reactive oxygen species (ROS) and excessive consumption of these enzymes (Yuan et al. 2020; Wu et al. 2020).

Natural products exert protective effects mainly through free radical scavenging mechanisms (Essawy et al. 2018). In this study, the administration of GbE and/or LC significantly reduced both MDA and NO levels and exhibited significant restoration of normal GSH levels and the antioxidant enzymatic activities. GbE and LC are well studied as antioxidant compounds in different experimental models. However, many reports have discussed the neuroprotective effect of GbE against brain-induced toxicity (Mohamed et al. 2020), and others illustrated the defensive role of LC in induced neuroinflammation and neurodegeneration models (Hussein et al. 2018), but there is no documented work on the effect of their combination. The neuroprotective and therapeutic effects of GbE and LC could be mediated via their antioxidant properties by directly scavenging ROS, decreasing lipid peroxidation, increasing the antioxidant system, or indirectly preventing free radical production, mitochondrial dysfunction, and inflammation (Abd-Elhady et al. 2013; Verma et al. 2019; Hamza et al. 2020).

AchE is one of the brain’s highly active enzymes that is widely expressed in different brain regions and has several functions. It is an enzyme that terminates the cholinergic transmission by rapid hydrolysis of the neurotransmitter acetylcholine (Ach). The current results showed an elevation in AchE activity in the hippocampus and hypothalamus of PTZ-kindled rats. This result agrees with the finding of Alachkar et al. (2020) who indicated that PTZ administration caused a significant elevation in the activity of AchE in the hippocampal tissues of PTZ-kindled rats. An elevation in AchE activity leads to a decrease in Ach levels and reduction of cholinergic neurotransmission efficiency, thus contributing to neurological dysfunctions (Essawy et al. 2019). According to Komali et al. (2021), AchE could be used as an index of cholinergic functions, and changes in the enzyme activity may indicate alterations in the availability of Ach at the level of its receptors.

On the other hand, pre- and post-treatment of kindled rats with either GbE or LC inhibited PTZ-induced elevation in AchE activity. Assaf et al. (2012) reported that LC mediates the transfer of acetyl group for Ach synthesis, as well as it could influence the signal transduction pathways and gene expression. Moreover, the neuroprotective property of GbE was attributed to the presence of flavonoids, terpenoids, and ginkgolic acids containing free radical scavengers (Kaur et al. 2013; Verma et al. 2019). This indicates the countervail actions of GbE and LC on cholinergic hyperexcitability via increasing the availability of Ach and acting synergistically with AchE inhibitors (Hamza et al. 2020; Komali et al. 2021).

Seizure activity is associated with a wide range of local biochemical changes, affecting various neurotransmitters such as monoamines and amino acids (Uddin et al. 2018). In the current study, PTZ was found to decrease NE, DA, and 5-HT levels in both the hippocampus and hypothalamus regions. These findings are concomitant with previous studies by Visweswari et al. (2010); Chimakurthy and Talasila (2010); and Abdel-Rahman et al. (2013). Monoamines play an essential role in the development and progression of epileptogenesis. The reduction in 5-HT level was found to be linked with a decrement in its synaptosomal uptake, the inhibition of tryptophan hydroxylase activity, and the decrease of tryptophan level in the epileptic model (Tchekalarova et al. 2011). Meanwhile, the reduced DA level in epileptic patients was attributed to the increased monoamine oxidase activity and the decreased reuptake (Rezaei et al. 2017). Furthermore, NE is a neuromodulator that acts as an anticonvulsant agent, and its decrease in epileptic patients is due to the downregulation of α1 receptors’ density in the brain tissue. This decrement could be also due to the decrease in dopamine-β-hydroxylase activity, the rate-limiting enzyme in NE synthesis (Yuan et al. 2020).

Pre- and post-treatment of kindled rats with either GbE or LC significantly alleviated the changes in monoamine (NE, DA, and 5-HT) levels in the rats’ hippocampus and hypothalamus. Similarly, Tousson et al. (2015) have reported that the administration of GbE (100 mg/kg) and LC (300 mg/kg) has improved the biochemical alterations in the cerebral cortex in a rat seizure model. These results reflect the potent anticonvulsant efficiency of GbE and LC as suggested by Ahmed and Mahmoud (2012) and Verma et al. (2019). Our findings lend more evidence to the hypothesis that the antiepileptic effect of GbE and LC could be partially related to their antioxidant effects. The modulating effect of GbE and LC on the studied monoamines might be due to the prevention of neuronal loss by suppressing ROS-mediated reactions through suppression of microglial activation and the associated release of oxygen intermediates and inflammatory factors. Moreover, Waggas and Al-Hasani (2010) showed that GbE causes a significant increase in the levels of neurotransmitters via the inhibition of calcium-ATPase and phosphodiesterase, as well as the blocking of Ca^2+^/calmodulin binding which play an important role in the release of the neurotransmitters.

Our histopathological investigations showed that administration of PTZ induced neuronal loss, gliosis, the appearance of dark neurons, vacuolar degeneration, and several other histopathological alterations in both hippocampal and hypothalamic regions. This result was consistent with previous studies indicating that seizure activity is associated with neuronal distortion and the appearance of necrotic and apoptotic forms of cells (Saha et al. 2014). These alterations have largely been attributed to excessive oxidative and nitrosative stress and neuroinflammation mediated by activated microglia.

Supplementation of GbE or LC in combination with PTZ showed marked improvement in the histological appearance of the hippocampal and hypothalamic neurons by suppressing ROS-mediated reactions. Abdou et al. (2016) attributed this improvement to the antiapoptotic effects, maintaining mitochondrial integrity and decreasing the caspase transcription rate and DNA fragmentation. According to Kaur et al. (2013), GbE, and some of its constituents, such as ginkgolide B and bilobalide, protected neurons against apoptotic and excitotoxic damage. Also, Babu et al. (2011) and Hazzaa et al. (2021) reported the neuroprotective role of LC through its antioxidant and anti-inflammatory effects.

In order to investigate the possibility of the involvement of astroglial activation in PTZ-induced epileptic seizures, the cellular expression of astroglia in the hippocampus and hypothalamus was examined by immunohistochemistry using ezrin protein, a marker protein for astroglia and ependymal cells in the CNS. In our results, the increased ezrin expression after PTZ administration indicates the activation of the astroglial cells suggesting that systemic PTZ treatment enhances astrocytosis in the brain. Treatment with GbE or LC induced a reduction in astrogliosis and a decrease in ezrin immunoreactivity. GbE exerts a neuroprotective effect via maintaining glutamate uptake by astrocytes and thus reducing excitotoxic injury (Rocher et al. 2011). Furthermore, LC may be mediated by its neuroprotective effect by astrocyte mitochondrial metabolism as the differences in metabolism between astrocytes and neurons affect their responses to neurodegeneration (Bambrick et al. 2004).

## Conclusions

The outcomes of our studies are of clinical importance, particularly the use of GbE and LC as safe neuroprotective and neurotherapeutic agents against neurotoxicity in the induced kindling seizures. The potential useful effects of GbE and LC could be via inhibition of free radical production, scavenging of ROS, and reactivation of antioxidant defense systems.

## Data Availability

All the data and material were available. The data of this article are included within the article and its additional files.
